# Associations between body mass index, waist circumference, waist-to-height ratio, and high blood pressure among adolescents: a cross-sectional study

**DOI:** 10.1038/s41598-019-45956-9

**Published:** 2019-07-01

**Authors:** Renata Kuciene, Virginija Dulskiene

**Affiliations:** Institute of Cardiology, Medical Academy, Lithuanian University of Health Sciences, Sukileliu 15, LT-50161 Kaunas, Lithuania

**Keywords:** Risk factors, Epidemiology, Hypertension

## Abstract

The purpose of the present study was to examine the associations between body mass index (BMI), waist circumference (WC), waist-to-height ratio (WHtR), and high blood pressure (HBP), and to determine which anthropometric parameters can best predict HBP among Lithuanian adolescents aged 12–15 years. Data from the survey of “Prevalence and Risk Factors of HBP in 12–15-Year-Old Lithuanian Children and Adolescents (Study 1, 2010–2012)” were used; a total of 7,457 respondents (3,494 boys and 3,963 girls) were included in this analysis. Adolescents with BP above the 90th percentile were measured on two different occasions. Logistic regression analysis was used to assess the associations and to calculate odds ratios. Receiver operating characteristic (ROC) curve analysis was used to evaluate the predictive ability of the three anthropometric parameters to predict HBP. The adjusted odds ratios (aOR) in the highest quartiles of BMI, WC, and WHtR were statistically significant for both sexes separately (reference group – the first quartile): in boys, prehypertension – 4.91, 4.09, and 1.59; hypertension – 7.96, 6.44, and 2.81; and prehypertension/hypertension – 6.85, 5.65, and 2.37, respectively; and in girls, prehypertension – 3.42, 2.70, and 1.66; hypertension – 5.71, 3.54, and 2.90; and prehypertension/hypertension – 4.62, 3.17, and 2.31, respectively). According to the analyses of the ROC curve, BMI z-score provided the largest area under the curve (AUC) value, followed by WC z-score, while WHtR z-score showed the lowest AUC value in predicting elevated BP in both sexes separately. Among Lithuanian adolescents aged 12–15 years, both anthropometric indices – BMI and WC (but particularly BMI) – showed stronger associations with HBP and were better for the prediction of HBP, compared to WHtR.

## Introduction

Hypertension (known as high or raised blood pressure) is one of the most common and important public health problems globally^1^. High blood pressure is associated with adverse cardiovascular outcomes^2^, and it is also considered to be the leading risk factor for mortality in the world, causing 7.5 million deaths per year, which accounts for 12.8% of all deaths^3^. The prevalence of raised BP rose from 594 million to 1.13 billion between 1975 and 2015 in people aged 18 years and older^4^. Epidemiological studies have reported a high prevalence of increased blood pressure in different age groups from childhood to adolescence in various countries^5–10^. Lithuania is no exception, where national research studies have showed high prevalence of high blood pressure (HBP) in Lithuanian preschoolers (21.4%)^11^ and children and adolescents (prehypertension – 12.8%, and hypertension – 22.2%)^12^. Moreover, in Lithuania, a high prevalence of hypertension was reported in Lithuanian adult population during the period of 25 years – from 1983 to 2008^13^. In addition, CVD incidence and mortality rates in our country are among the highest in Europe^14^.

Systematic reviews and meta-analyses demonstrated that BP levels persist from childhood to adulthood^15,16^. Hypertension during puberty is a powerful predictor of adult hypertension^17^. Many various interrelated genetic, metabolic, environmental, behavioral, psychosocial, and socioeconomic risk factors as well as family and personal medical history may influence hypertension in adolescents^18^. Early atherosclerotic lesions, left ventricular hypertrophy, an increase in the carotid artery intima-media thickness, retinal vascular changes, and cognitive function disorders are detected in children with HBP^19^. The early identification of HBP in children may prevent the development and progression of cardiovascular diseases and their complications^20^.

General^21^ and abdominal obesity^22^ in childhood and adolescence is a serious growing health problem worldwide. According to the Non-Communicable Disease Risk Factor Collaboration (NCD-RisC), from 1975 to 2016, among children and adolescents aged 5–19 years, the number of obese boys increased from 6 (1–19) millions to 74 (39–125) millions, and the number of girls with obesity increased from 5 (1–14) millions to 50 (24–89) millions^23^. The systematic review of twenty-nine studies showed that the prevalence of abdominal obesity among adolescents aged 10–19 years ranged from 3.8 to 51.7% in low-to-middle-income countries and from 8.7 to 33.2% in developed countries^24^.

Obesity in childhood and adolescence is associated with higher risk of cardiovascular, metabolic, and endocrine disorders (hypertension, dyslipidemia, endothelial dysfunction, chronic inflammation, the metabolic syndrome, type 2 diabetes mellitus, and pubertal disorders)^25^, renal, gastrointestinal, pulmonary, musculoskeletal, dermatologic, neurologic, and psychosocial disorders^26^. The data from a study of the markers of subclinical atherosclerosis showed that obese children and adolescents have significantly increased carotid artery intima-media thickness levels, and higher serum levels of biomarkers of inflammation, as compared with non-obese participants^27^. Abdominal obesity in children is also related to multiple adverse cardiometabolic risk factors such as hypertension, the metabolic syndrome, lipid abnormalities, glucose intolerance, and insulin resistance, which contribute to an increased risk of developing atherosclerosis^28^. Childhood and adolescence obesity tracks into adulthood^29^ and is associated with adult cardiovascular morbidity and mortality^30^.

In research settings and in clinical practice, the most commonly used surrogate measures of general obesity and abdominal obesity could predict the risk of cardiometabolic outcomes, cardiovascular disease and all-cause mortality^31^. The systematic review analysis indicated that WHtR ≥ 0.5 is a predictor of cardiovascular diseases and diabetes in adult and children populations^32^. A meta-analysis including thirty-four cross-sectional studies with a total of 169,630 children and adolescents showed that WHtR was not superior to BMI and WC in screening for cardio-metabolic risk factors^33^. However, it remains unclear which of these anthropometric indices are the best predictors of HBP in adolescents. Furthermore, there is limited scientific evidence regarding the prediction of prehypertension by anthropometric parameters. Consequently, the present study focused on prehypertension and hypertension analyzed separately and in combination among children and adolescents. The relationships between WHtR and prehypertension and hypertension have not been studied among Lithuanian schoolchildren before.

The aim of the study was to examine the associations between BMI, WC, WHtR, and prehypertension and hypertension, and to determine which of these anthropometric indices are the best predictors of HBP among Lithuanian adolescents aged 12–15 years.

## Results

The final study sample consisted of 7,457 participants (46.9% were boys, and 53.1% were girls) with a median age of 13.43 ± 9.93 years (Table 1). No differences in the median age were found between the sexes. Median values of weight, height, BMI, WC, WHtR, and body roundness index (BRI) were higher in boys than in girls. Boys also had higher median values of SBP (systolic blood pressure), MAP (mean arterial pressure), and PP (pulse pressure) than girls did. The median value of DBP (diastolic blood pressure) was higher in girls than boys. The prevalence of prehypertension was 15.0% in boys and 10.9% in girls, while the prevalence of hypertension was 29.1% in boys and 16.1% in girls.

**Table 1:** Characteristics of the study participants by sex; the values are presented as median (25th–75th percentiles).

Variables	Boys (n = 3494)	Girls (n = 3963)	p*
Age (years)	13.0 (12.0–14.0)	13.0 (12.0–14.0)	0.924
Weight (kg)	54.35 (45.0–64.0)	51.0 (45.0–58.0)	<0.001
Height (cm)	167.0 (158.0–175.0)	163.0 (158.0–168.0)	<0.001
BMI (kg/m^2^)	19.23 (17.48–21.26)	19.0 (17.30–21.01)	0.001
WC (cm)	68.0 (64.0–73.0)	64.0 (60.5–68.0)	<0.001
WHtR	0.41 (0.39–0.43)	0.39 (0.37–0.42)	<0.001
BRI	1.79 (1.48–2.20)	1.58 (1.28–1.97)	<0.001
SBP (mm Hg)	117.67 (110.67–133.33)	114.0 (107.3–120.67)	<0.001
DBP (mm Hg)	64.33 (59.67–70.00)	65.33 (60.67–70.67)	<0.001
MAP (mm Hg)	82.89 (77.44–89.44)	81.56 (76.78–87.11)	<0.001
PP (mm Hg)	54.33 (46.33–65.67)	48.33 (42.67–54.67)	<0.001

*Boys versus girls.

BP – blood pressure, BMI – body mass index, WC – waist circumference, WHtR – waist-to-height ratio, BRI – body roundness index, SBP – systolic blood pressure, DBP – diastolic blood pressure, MAP – mean arterial pressure, PP – pulse pressure.

The comparison of the subjects with NBP and with HBP (prehypertension and hypertension) revealed statistically significant differences in anthropometric indices in both sexes separately (Table 2). The number of cases and the prevalence of HBP increased with increasing quartiles of all anthropometric parameters in both sexes (the first quartile vs. the fourth quartile). For prehypertension, the data were the following: BMI, 3.3% vs. 4.0% for boys and 3.0% vs. 3.3% for girls; WC, 2.2% vs. 5.1% for boys and 1.6% vs. 4.0% for girls; and WHtR, 3.7% vs. 3.9% for boys and 2.4% vs. 3.2% for girls. For hypertension, the data were the following: BMI, 5.4% vs. 10.0% for boys and 3.4% vs. 5.6% for girls; WC, 3.1% vs. 11.6% for boys and 2.0% vs. 6.6% for girls; and WHtR, 5.6% vs. 10.3% for boys and 2.6% vs. 6.2% for girls. The subjects (boys and girls separately) with HBP demonstrated significantly higher median values of all analyzed variables, compared to normotensive participants (Table 3). The median values of all anthropometric variables except height, and the median values of BP (SBP, DBP, MAP, and PP) increased with increasing quartiles of BMI, WC, and WHtR. The highest median values of SBP, DBP, MAP, and PP were found in participants in the highest (fourth) quartiles of anthropometric indices, especially BMI (data not shown).

**Table 2:** Characteristics of the study participants according to BP level.

Variables	Normotensive	Prehypertensive	Hypertensive	p*
***Boys***				
**Quartiles of BMI:**				
1^st^	1016 (52.0)	115 (21.9)^§^	189 (18.6)^§^	<0.001
2^nd^	409 (21.0)	118 (22.6)	185 (18.1)^#^	
3^rd^	315 (16.1)	151 (28.8)^§^	295 (29.0)^§^	
4^th^	212 (10.9)	140 (26.7)^§^	349 (34.3)^§,#^	
**Quartiles of WC:**				
1^st^	574 (29.4)	78 (14.9)^§^	110 (10.8)^§,#^	<0.001
2^nd^	521 (26.7)	126 (24.0)	230 (22.6)^§^	
3^rd^	514 (26.3)	143 (27.3)	274 (26.9)	
4^th^	343 (17.6)	177 (33.8)^§^	404 (39.7)^§,#^	
**Quartiles of WHtR:**				
1^st^	544 (27.9)	128 (24.4)	195 (19.1)^§,#^	<0.001
2^nd^	519 (26.6)	129 (24.6)	229 (22.5)^§^	
3^rd^	511 (26.1)	130 (24.8)	233 (22.9)^§^	
4^th^	378 (19.4)	137 (26.2)^§^	361 (35.5)^§,#^	
Weight (kg)	49.0 (41.0–56.0)	60.0 (54.0–68.0)^a^	61.0 (54.0–70.0)^a^	<0.001
Height (cm)	162.0 (155.0–170.0)	173.5 (167.0–179.38)^a^	172.0 (164.0–178.0)^a,b^	<0.001
BMI (kg/m^2^)	18.24 (16.88–19.96)	20.14 (18.6–21.94)^a^	20.76 (18.94–22.80)^a,b^	<0.001
WC (cm)	66.0 (62.0–70.0)	70.0 (66.0–75.0)^a^	71.0 (67.0–77.0)^a,b^	<0.001
WHtR	0.41 (0.38–0.43)	0.41 (0.38–0.43)	0.42 (0.39–0.45)^a,b^	<0.001
BRI	1.76 (1.45–2.11)	1.76 (1.45–2.19)	1.91 (1.55–2.44)^a,b^	<0.001
SBP (mm Hg)	111.67 (105.67–115.33)	126.67 (123.0–129.25)^a^	140.0 (135.0–146.33)^a,b^	<0.001
DBP (mm Hg)	62.0 (58.33–66.33)	65.33 (60.67–70.0)^a^	69.33 (64.33–75.67)^a,b^	<0.001
MAP (mm Hg)	78.33 (74.56–82.11)	85.44 (82.36–88.89)^a^	93.56 (89.0–98.33)^a,b^	<0.001
PP (mm Hg)	47.67 (42.33–52.67)	60.33 (56.08–65.92)^a^	71.0 (64.67–78.0)^a,b^	<0.001
***Girls***				
**Quartiles of BMI:**				
1^st^	1321 (45.6)	119 (27.5)^§^	135 (21.2)^§,#^	<0.001
2^nd^	648 (22.4)	83 (19.3)	127 (19.9)	
3^rd^	543 (18.8)	99 (23.0)^§^	154 (24.2)^§^	
4^th^	383 (13.2)	130 (30.2)^§^	221 (34.7)^§^	
**Quartiles of WC:**				
1^st^	695 (24.0)	62 (14.4)^§^	78 (12.2)^§^	<0.001
2^nd^	755 (26.1)	78 (18.1)^§^	110 (17.3)^§^	
3^rd^	783 (27.0)	132 (30.6)	186 (29.2)	
4^th^	662 (22.9)	159 (36.9)^§^	263 (41.3)^§^	
**Quartiles of WHtR:**				
1^st^	770 (26.7)	95 (22.0)^§^	104 (16.3)^§,#^	<0.001
2^nd^	780 (26.9)	96 (22.3)^§^	126 (19.8)^§^	
3^rd^	722 (24.9)	113 (26.2)	163 (25.6)	
4^th^	623 (21.5)	127 (29.5)^§^	244 (38.3)^§,#^	
Weight (kg)	50.0 (43.0–55.50)	56.0 (50.0–63.0)^a^	55.5 (49.0–63.0)^a^	<0.001
Height (cm)	163.0 (157.0–168.0)	166.0 (162.0–171.0)^a^	164.0 (159.0–169.0)^a,b^	<0.001
BMI (kg/m^2^)	18.59 (16.94–20.32)	20.07 (18.16–22.31)^a^	20.54 (18.68–23.12)^a,b^	<0.001
WC (cm)	63.0 (60.0–67.0)	66.0 (62.0–71.0)^a^	66.0 (63.0–72.0) ^a^	<0.001
WHtR	0.39 (0.37–0.41)	0.40 (0.38–0.43)^a^	0.41 (0.38–0.44)^a,b^	<0.001
BRI	1.53 (1.25–1.89)	1.64 (1.32–2.07)^a^	1.77 (1.41–2.34)^a,b^	<0.001
SBP (mm Hg)	110.67 (105.0–114.67)	123.0 (121.33–125.33)^a^	134.0 (129.67–139.83)^a,b^	<0.001
DBP (mm Hg)	63.67 (59.33–67.67)	69.00 (65.0–74.0)^a^	74.33 (69.33–79.17)^a,b^	<0.001
MAP (mm Hg)	79.11 (75.33–82.78)	87.44 (84.22–90.67)^a^	94.22 (90.11–98.78)^a,b^	<0.001
PP (mm Hg)	45.33 (41.0–50.33)	54.0 (49.67–58.67)^a^	61.0 (55.67–67.0)^a,b^	<0.001
***Boys***				
**Age (years):**				
12–13	1247 (63.9)	151 (28.8)^§^	413 (40.6)^§,#^	<0.001
14–15	705 (36.1)	373(71.2)^§^	605 (59.4)^§,#^	
	13.0 (12.0–14.0)	14.0 (13.0–15.0)^a^	14.0 (13.0–15.0)^a,b^	<0.001
***Girls***				
**Age (years):**				
12–13	1576 (54.4)	171 (39.7)^§^	324 (50.9)^#^	<0.001
14–15	1319 (45.6)	260 (60.3)^§^	313 (49.1)^#^	
	13.0 (12.0–14.0)	14.0 (13.0–15.0)^a^	13.0 (12.5–14.0)^a,b^	<0.001

The values are numbers (percentages) and median (25th–75th percentiles).

The chi-square (χ^2^) test was used for categorical variables.

^§^P < 0.05 vs. NBP group (z test).

^#^P < 0.05 vs. prehypertension group (z test).

^a^P < 0.05 vs. NBP group.

^b^P < 0.05 vs. prehypertension group.

BMI – body mass index, WC – waist circumference, WHtR – waist-to-height ratio, BRI – body roundness index, SBP – systolic blood pressure, DBP – diastolic blood pressure, MAP – mean arterial pressure, PP – pulse pressure.

**Table 3:** Pearson’s correlation coefficients between anthropometric parameters z-scores and blood pressure.

		BMI z-score	WC z-score	WHtR z-score
SBP (mm Hg)	Boys	0.404**	0.387**	0.133**
	Girls	0.366**	0.305**	0.205**
DBP (mm Hg)	Boys	0.209**	0.219**	0.122**
	Girls	0.207**	0.185**	0.115**
MAP (mm Hg)	Boys	0.352**	0.348**	0.146**
	Girls	0.305**	0.262**	0.170**
PP (mm Hg)	Boys	0.355**	0.328**	0.087**
	Girls	0.308**	0.246**	0.173**

**Correlation is significant at the level of 0.01 (2-tailed).

BMI – body mass index, WC – waist circumference, WHtR – waist-to-height ratio, SBP – systolic blood pressure, DBP – diastolic blood pressure, MAP – mean arterial pressure, PP – pulse pressure.

Pearson’s correlation coefficients between anthropometric indexes z-scores and BP are shown in Table 3. BMI z-score, WC z-score, and WHtR z-score positively and significantly correlated with BP in boys and in girls, but the strongest correlations found for BP were with BMI z-score and WC z-score. In particular, the highest correlations were found between BMI z-score and SBP and between WC z-score and SBP in boys, and between BMI z-score and SBP and PP in girls.

SBP correlated significantly with DBP (for boys: r = 0.526, p < 0.001; for girls: r = 0.647, p < 0.001). Strong correlations were found between MAP and SBP (for boys: r = 0.877, p < 0.001; for girls: r = 0.883, p < 0.001) and DBP (for boys: r = 0.870, p < 0.001; for girls: r = 0.929, p < 0.001).

Correlation coefficients between BMI z-score and WC z-score (r = 0.774 for boys and r = 0.793 for girls), between BMI z-score and WHtR z-score (r = 0.660 for boys and r = 0.725 for girls), and between WC z-score and WHtR z-score (r = 0.800 for boys and r = 0.894 for girls) were positive and statistically significant (all p < 0.001).

In both sexes, aORs increased with the increasing quartile of BMI, WC, and WHtR (Table 4). Adjusted odds ratios in the highest quartiles of BMI, WC, and WHtR were statistically significant in boys (girls): prehypertension – 4.91 (3.42), 4.09 (2.70), and 1.59 (1.66); hypertension – 7.96 (5.71), 6.44 (3.54), and 2.81 (2.90); and prehypertension/hypertension – 6.85 (4.62), 5.65 (3.17), and 2.37 (2.31), respectively. The increase in aORs by BMI quartiles was higher than the respective increase by WC quartiles (except for aOR for prehypertension in the third quartile among girls). The odds ratios were the lowest in WHtR quartiles. In boys, statistically significant aORs for HBP were detected in the fourth quartile of WHtR and for hypertension – in the third quartile of WHtR. In boys, no significant associations were observed for prehypertension or prehypertension/hypertension in the second or the third quartiles of WHtR. In girls, no significant associations were found for any HBP categories in the second quartiles of WC and WHtR, and no significant associations for prehypertension were found in the third quartiles of WHtR. The models with BMI had the lowest values of AIC; then, in the ascending order of AIC values, followed models with WC and WHtR.

**Table 4:** Crude and adjusted odds ratios and 95% confidence intervals for HBP in quartiles of anthropometric parameters (BMI, WC, WHtR) by sex (univariate and multivariate analyses).

Variables	Prehypertension		Hypertension		Prehypertension/Hypertension	
	OR (95% CI)	aOR(95% CI)	OR (95% CI)	aOR(95% CI)	OR (95% CI)	aOR(95% CI)
**Boys:**						
**Quartiles of BMI:**						
1^st^	1.00	1.00	1.00	1.00	1.00	1.00
2^nd^	**2**.**55 (1**.**92–3**.**38)**	**1**.**99 (1**.**49–2**.**66)**	**2**.**43 (1**.**93–3**.**07)**	**2**.**12 (1**.**67–2**.**69)**	**2**.**48 (2**.**04–3**.**01)**	**2**.**10 (1**.**72–2**.**57)**
3^rd^	**4**.**24 (3**.**22–5**.**57)**	**3**.**29 (2**.**48–4**.**37)**	**5**.**03 (4**.**03–6**.**29)**	**4**.**28 (3**.**41–5**.**37)**	**4**.**73 (3**.**90–5**.**74)**	**3**.**90 (3**.**20–4**.**76)**
4^th^	**5**.**83 (4**.**38–7**.**78)**	**4**.**91 (3**.**64–6**.**62)**	**8**.**85 (7**.**03–11**.**15)**	**7**.**96 (6**.**30–10**.**06)**	**7**.**71 (6**.**27–9**.**47)**	**6**.**85 (5**.**55–8**.**46)**
**Quartiles of WC:**						
1^st^	1.00	1.00	1.00	1.00	1.00	1.00
2^nd^	**1**.**78 (1**.**31–2**.**42)**	**1**.**89 (1**.**37–2**.**59)**	**2**.**30 (1**.**78–2**.**98)**	**2**.**31 (1**.**78–3**.**00)**	**2**.**09 (1**.**69–2**.**58)**	**2**.**13 (1**.**70–2**.**65)**
3^rd^	**2**.**05 (1**.**52–2**.**77)**	**2**.**32 (1**.**70–3**.**17)**	**2**.**78 (2**.**16–3**.**58)**	**2**.**98 (2**.**30–3**.**85)**	**2**.**48 (2**.**01–3**.**05)**	**2**.**75 (2**.**21–3**.**41)**
4^th^	**3**.**80 (2**.**82–5**.**12)**	**4**.**09 (2**.**99–5**.**58)**	**6**.**15 (4**.**79–7**.**89)**	**6**.**44 (4**.**98–8**.**32)**	**5**.**17 (4**.**18–6**.**39)**	**5**.**65 (4**.**53–7**.**04)**
**Quartiles of WHtR:**						
1^st^	1.00	1.00	1.00	1.00	1.00	1.00
2^nd^	1.06 (0.80–1.39)^NS^	1.08 (0.81–1.43)^NS^	1.23 (0.98–1.54)^NS^	1.25 (0.99–1.58)^NS^	1.16 (0.96–1.41)^NS^	1.17 (0.96–1.44)^NS^
3^rd^	1.08 (0.82–1.42)^NS^	1.13 (0.85–1.50)^NS^	**1**.**27 (1**.**02–1**.59)***	**1**.**27 (1**.**01–1**.61)***	1.20 (0.99–1.45)^NS^	1.22 (0.99–1.48)^NS^
4^th^	**1**.**54 (1**.**17–2**.03)**	**1**.**59 (1**.**19–2**.12)**	**2**.**66 (2**.**14–3**.**31)**	**2**.**81 (2**.**24–3**.**52)**	**2**.**22 (1**.**83–2**.**69)**	**2**.**37 (1**.**94–2**.**90)**
**Girls**:						
**Quartiles of BMI:**						
1^st^	1.00	1.00	1.00	1.00	1.00	1.00
2^nd^	**1**.**42 (1**.**06–1**.91)***	1.32 (0.98–1.77)^NS^	**1**.**92 (1**.**48–2**.**49)**	**1**.**94 (1**.**49–2**.**51)**	**1**.**69 (1**.**37–2**.**07)**	**1**.**64 (1**.**33–2**.**02)**
3^rd^	**2**.**02 (1**.**52–2**.**69)**	**1**.**86 (1**.**39–2**.**47)**	**2**.**78 (2**.**16–3**.**57)**	**2**.**80 (2**.**17–3**.**62)**	**2**.**42 (1**.**98–2**.**96)**	**2**.**35 (1**.**92–2**.**88)**
4^th^	**3**.**77 (2**.**87–4**.**95)**	**3**.**42 (2**.**59–4**.**52)**	**5**.**65 (4**.**43–7**.**20)**	**5**.**71 (4**.**47–7**.**29)**	**4**.**77 (3**.**91–5**.**81)**	**4**.**62 (3**.**79–5**.**64)**
**Quartiles of WC:**						
1^st^	1.00	1.00	1.00	1.00	1.00	1.00
2^nd^	1.16 (0.82–1.64)^NS^	1.17 (0.82–1.66)^NS^	1.30 (0.95–1.77)^NS^	1.30 (0.96–1.77)^NS^	1.24 (0.97–1.57)^NS^	1.24 (0.97–1.58)^NS^
3^rd^	**1**.**89 (1**.**37–2**.**60)**	**1**.**89 (1**.**37–2**.**60)**	**2**.**12 (1**.**59–2**.**81)**	**2**.**11 (1**.**59–2**.**81)**	**2**.**02 (1**.**61–2**.**52)**	**2**.**01 (1**.**61–2**.**52)**
4^th^	**2**.**69 (1**.**97–3**.**68)**	**2**.**70 (1**.**97–3**.**69)**	**3**.**54 (2**.**69–4**.**66)**	**3**.**54 (2**.**69–4**.**66)**	**3**.**17 (2**.**54–3**.**94)**	**3**.**17 (2**.**55–3**.**95)**
**Quartiles of WHtR:**						
1^st^	1.00	1.00	1.00	1.00	1.00	1.00
2^nd^	0.99 (0.74–1.35)^NS^	0.99 (0.73–1.34)^NS^	1.20 (0.91–1.58)^NS^	1.20 (0.91–1.58)^NS^	1.10 (0.89–1.37)^NS^	1.10 (0.88–1.36)^NS^
3^rd^	1.27 (0.95–1.70)^NS^	1.27 (0.95–1.70)^NS^	**1**.**67 (1**.**28–2**.**18)**	**1**.**67 (1**.**28–2**.**18)**	**1**.**48 (1**.**20–1**.**82)**	**1**.**48 (1**.**20–1**.**82)**
4^th^	**1**.**65 (1**.**24–2**.20)**	**1**.**66 (1**.**25–2**.21)**	**2**.**90 (2**.**25–3**.**73)**	**2**.**90 (2**.**26–3**.**74)**	**2**.**30 (1**.**88–2**.**82)**	**2**.**31 (1**.**89–2**.**83)**

OR – crude odds ratio; aOR^1^ – adjusted odds ratios for age; CI – confidence interval.

Bold typeface indicates significance. All results were significant at P < 0.001, except when noted ((**P < 0.01; ***P < 0.05; NS – not significant).

BMI – body mass index, WC – waist circumference, WHtR – waist-to-height ratio.

Table 5 presents the results from ROC analysis for BMI, WC, and WHtR for the prediction of HBP for each sex separately. BMI z-score showed the highest AUC value, followed by WC z-score, while WHtR z-score provided the lowest AUC value for predicting elevated BP (either alone or in combination) in both boys and girls. The AUC value for predicting hypertension and prehypertension/hypertension was higher than the AUC value for predicting prehypertension. The AUC values of BMI z-score and WC z-score were greater in boys than in girls, while the opposite was found for the WHtR z-score.

**Table 5:** Area under ROC curves (95% CI) of anthropometric indices to predict elevated BP.

Variables	Prehypertension	Hypertension	Prehypertension/hypertension
**Boys**			
BMI z-score	0.699 (0.675–0.724)*	0.741 (0.722–0.759)*	0.727 (0.710–0.743)*
WC z-score	0.694 (0.670–0.718)*	0.719 (0.700–0.738)*	0.711 (0.694–0.728)*
WHtR z-score	0.513 (0.485–0.542)*	0.584 (0.562–0.606)*	0.560 (0.541–0.579)*
**Girls**			
BMI z-score	0.650 (0.622–0.677)*	0.690 (0.668–0.713)*	0.674 (0.655–0.693)*
WC z-score	0.637 (0.609–0.664)*	0.652 (0.628–0.676)*	0.646 (0.627–0.665)*
WHtR z-score	0.560 (0.530–0.589)*	0.622 (0.597–0.647)*	0.597 (0.577–0.617)*

Data are AUC (95% confidence interval).

*P value < 0.001.

AUC – area under the receiver operating characteristic curve, BMI – body mass index, WC – waist circumference, WHtR – waist-to-height ratio.

## Discussion

In our study, we found a high prevalence of prehypertension (12.8%) and hypertension (22.2%) in Lithuanian adolescents aged 12–15 years, which is partially in line with findings from other studies performed in different populations of children and adolescents in other countries, for example, in 9–13 year-old Greek schoolchildren (prehypertension – 14.2% and hypertension – 23%)^7^, in Chinese schoolchildren aged 5 to 18 years (prehypertension – 15.2% and hypertension – 20.5%)^6^, in Portuguese children and adolescents aged 4 to 18 years (prehypertension – 21.6% and hypertension – 12.8%)^5^, in Spanish children aged 4 to 6 years (prehypertension – 12.3% and hypertension – 18.2%)^8^, in 11–14 year-old Italian schoolchildren (prehypertension – 10.3% and hypertension – 10.1%)^34^, or in South African adolescents aged 13–17 years (prehypertension – 12.3% and hypertension – 21.3%)^35^. However, differences in the times of BP visits, BP measurement methods (the auscultatory method or the oscillometric technique), sample size, the age of the examined children and adolescents, and disparities across ethnicity, socioeconomic status, and different geographic regions between the studies make comparison of the results difficult. Nevertheless, epidemiologic data suggest that HBP is an important and common health problem among adolescents; therefore, it is essential to develop and implement effective public health strategies to prevent and to control prehypertension and hypertension. Early identification, control and treatment of modifiable risk factors, and healthy lifestyle changes (particularly in children and adolescents) may reduce the risk of cardiovascular diseases and other chronic non-communicable diseases and may prevent a large disease burden in the future. It is also important to focus attention on subjects with established prehypertension or hypertension – with either high risk or very high risk of cardiometabolic comorbidities. However, taking into account the recommendations and guidelines used for the evaluation and treatment of HBP in children and adolescents, it can often be underdiagnosed. For instance, in a large cohort study of pediatric population, a high frequency of undiagnosed prehypertension and hypertension was found^36^. There is suggestive evidence that both prehypertension and hypertension in adolescents and youth are significant determinants of cardiovascular target organ damage^37^, and these adverse changes are strongly related to an increased risk of cardiovascular events in adulthood^38^. The analysis of a meta-analysis of prospective studies demonstrated that prehypertension was associated with a higher risk of incident stroke, myocardial infarction, and total cardiovascular outcomes^39^.

In the present study, BMI z-score, WC z-score, and WHtR z-score significantly correlated with SBP, DBP, MAP, and PP. However, the correlations of WHtR z-score with BP were weaker than the correlations of BMI z-score and WC z-score. The aORs for HBP in BMI quartiles were higher than in WC quartiles, but were the lowest in WHtR quartiles. The aORs were significant in fourth quartiles of WHtR in both sexes. Significant associations were found in the second, third, and fourth quartiles of BMI and WC among boys. In girls, the associations with prehypertension in the second quartile of BMI and with either of the elevated BP levels in second quartiles of WC were not statistically significant. Meanwhile, other studies reported slightly different results. Silva *et al*. performed a study on Brazilian adolescents aged 14–19 years and found that elevated BP was significantly associated with both central and general obesity only in boys, but not in girls, comparing the fourth with the first quartile of the WC (≤69 cm vs. ≥80.1 cm) and BMI (≤18.6 kg/m^2^ vs. ≥23.5 kg/m^2^) (aOR = 6.97 and aOR = 6.44, repectively), while aORs for the second and the third quartiles were not significant after adjustment for age in a multivariate analysis^40^. In NHANES (National Health and Nutrition Examination Survey) (1988–2008), BMI (the third vs. the first quartile, OR = 1.43; and the fourth vs. the first quartile, OR = 2.00) and WC (the fourth vs. the first quartile, OR = 2.14) were significantly associated with an increased risk of elevated BP in children and adolescents aged 8 to 17 years after adjustment for age and sex^9^. Data from a cross-sectional study among 6–7 year-old children in Taiwan showed that in the combined group of boys and girls, high WC was significantly associated with HBP (aORs were 1.78, 2.45, and 6.03 in the second, third, and fourth quartiles of WC)^41^. A study that included Taiwanese children aged 7 years found that aORs of elevated BP, elevated SBP, and elevated DBP were significant in the second, third, and fourth quartiles of WHtR^42^.

In the current study, the ROC analysis showed that BMI z-score had the highest AUC value (0.727 for boys and 0.674 for girls) and was also a strong predictor of HBP, while WC z-score had a slightly lower AUC value (0.711 for boys and 0.646 for girls) compared to BMI z-score. The AUC value for WHtR z-score was the lowest among the three anthropometric indices (0.560 for boys and 0.597 for girls). Our findings are consistent with those of previous epidemiological studies conducted among children. A school-based cross-sectional survey among Chinese children aged 7–15 years found that BMI (AUC 0.74 for boys and 0.69 for girls) and WC (AUC 0.72 for boys and 0.66 for girls) were better predictors of elevated BP than WHtR were (AUC 0.69 for boys and 0.64 for girls)^43^. A study on 10–18 year-old adolescents from Tehran showed that BMI, after adjustment for sex and physical activity, was a better predictor of hypertension (AUC = 0.780) compared to WC (AUC = 0.739) or WHtR (AUC = 0.701)^44^. The findings of the current study are also in agreement with the results of a systematic review and meta-analysis of studies that included 25,424 children and adolescents aged 6 to 18 years and assessed the performance of obesity indices in identifying HBP. These results demonstrated that the AUCs for BMI, WC, and WHtR were 0.7780, 0.7181, and 0.6697, respectively^45^. In another cross-sectional population-based study performed in 99,366 Chinese children and adolescents aged 7–17 years, BMI in both sexes was a better predictor of HBP (with the AUCs being 0.656 in boys and 0.644 in girls) than other studied adiposity indicators (such as weight, WC, WHtR, hip circumference, waist-to-hip ratio, body adiposity index, and skin fold thickness)^46^. However, other studies have reported different findings. A study conducted in Indian schoolchildren aged 6–16 years showed that the AUC value of WHtR for high SBP was slightly higher than that of WC and BMI, while the AUC value of BMI for high DBP was slightly higher than that of WHtR and WC^47^. In a study conducted in Switzerland, Chiolero *et al*. found that BMI and WHtR alone and in combination had a similar and weak predictive ability to identify subjects with HBP in a group of children aged 10–14 years^48^. The interpretation and comparison of the results of associations and predictions among studies have been complicated, since there are differences regarding sample size, the age of the investigated subjects, the methodology of measurements, racial and ethnic criteria, and the potential confounders.

The results of our study also revealed that compared to WHtR, both anthropometric measures – BMI and WC (but especially BMI) – showed a stronger association with HBP and both were better predictors of HBP for both boys and girls. Both BMI and WC can be used to assess cardiovascular risk in children and adolescents in Lithuania. WC measurement has not yet been adopted or performed in clinical practice in our country; moreover, there are no national specific reference values and cutoff values of WC for children and adolescents. Epidemiological studies have found that children with low BMI but large WC may have a higher risk of HBP^49,50^. Moreover, adolescents with abnormal WC in any different BMI groups have increased odds of having elevated BP and abnormal cholesterol, glucose, triglyceride, and high-density lipoprotein levels^51^. The present study confirmed earlier observations by other researchers that the use of both BMI and WC is more effective than either measurement alone in identifying the risk of HBP^49,50^. The studies have reported that both BMI and WC were associated with HBP among children and adolescents^49,52^. Data from a multicenter cohort study (the German/Austrian/Swiss Adiposity Patients Registry) of adolescents aged 11–18 years revealed that BMI and WC were superior to WHtR in predicting obesity-related cardiometabolic risk^53^.

BMI, WC, and WHtR are easy, quick, noninvasive, simply obtainable and inexpensive measurements for predicting the risk of cardiovascular diseases^31^. BMI cannot distinguish between lean mass and body fat mass^54^. WC and WHtR cannot differentiate visceral from subcutaneous fat tissue^55^. WC measurement, in contrast to WHtR, does not account for height differences, as subjects with a similar WC but different height are not at the same risk for cardiometabolic risk factors^56^. Thus, there is no international agreement or a standard for accepted waist circumference cutoff values (which vary depending on age, sex, ethnicity, and race) for defining abdominal obesity among children and adolescents. Different WC measurement methods can result in different WC values^57^. During childhood and adolescence, the growth rates differ due to various factors (sex, age, the onset of puberty, and other factors), WC and height may increase differently and not in parallel within a subject, and the WHtR ratio changes and varies during these periods of growth and development^58^. It has been reported that the value of WHtR 0.5 indicates elevated health risks for children and adults^59^. Meanwhile, according to a review of the studies, WHtR cutoff value of 0.5 may be used for defining abdominal obesity and for predicting higher cardiometabolic risk in children aged 6 years and above, independent of sex, age, or ethnicity^60^. However, studies conducted in children and adolescents have showed that WHtR cutoff value of less than 0.5 can predict an increased risk for the development of high blood pressure, and in children and adolescents, hypertension can be identified in subjects with lower WHtR^61,62^. Our study suggests that subjects with the value of WHtR below 0.5 are at an increased risk of HBP, and the fourth quartile of WHtR was a risk factor for prehypertension and hypertension, both combined and separated.

In a research by Brambilla *et al*., according to the analysis of magnetic resonance imaging data, BMI has been found to be a better predictor of visceral adipose tissue, while WC was a better predictor of subcutaneous adipose tissue in subjects aged 7–16 years^63^. Barreira *et al*. analyzed the relationships between anthropometric parameters and fat mass and abdominal adiposity (based on the results of magnetic resonance imaging and dual energy X-ray absorptiometry) in subjects aged 5–18 years and found that WC and WHtR related with visceral adipose tissue (independent of sex and race). However, they more strongly related with subcutaneous adipose tissue and fat mass (dependent on sex and race)^64^. The Framingham Heart Study reported that both subcutaneous and visceral adipose tissues were associated with adverse metabolic risk factors, visceral adipose tissue being associated more strongly^65^. Meta-analysis showed that both BMI and WHtR strongly correlated with body fat (assessed by dual-energy X-ray absorptiometry) in children^66^. Body fat percentage and fat mass index are significantly associated with cardiovascular and metabolic risk factors^67^.

The mechanism of the association between obesity and hypertension may be explained by adipose tissue dysfunction characterized by decreased levels of adiponectin, hyperleptinemia, increased infiltration of macrophages, elevated free fatty acid levels, and elevated resistin levels, which leads to the activation of the sympathetic nervous system and the renin-angiotensin-aldosterone system, augmented systemic inflammation and oxidative stress, and chronic vascular inflammation, leading to hypertension^68^.

The current study has several limitations. In our study, BP was measured using a clinically validated automatic oscillometric device; schoolchildren with HBP were screened on two separate occasions within a period of 2–3 weeks. However, according to the Fourth Report^69^, HBP (exceeding the 90^th^ percentile) obtained by an oscillometric device should be repeated by auscultation, and, in addition, for confirming the diagnosis of hypertension, the measurement should be repeated on at least three separate occasions. In our research, biochemical parameters and pubertal status of the subjects were not evaluated. In addition, there was no adjustment for socioeconomic factors, family history of hypertension, and dietary factors since information on these potential confounding factors was lacking. In addition, in our research we included a relatively narrow age group of the schoolchildren population – only adolescents aged 12–15 years. Further studies are needed to examine the prevalence of HBP and to investigate the associations in children and adolescent populations across all age groups. The design of our study is cross-sectional, and therefore, causality cannot be determined. In an observational study, confounding, selection bias, and measurement or information bias can influence the results^70^.

In Lithuania, public health strategies should focus more on the understanding and prevention of cardiovascular disease risk factors. The results of our study would be useful in preparing preventive programs for improving children’s health. Healthy lifestyle changes and correction of adverse lifestyle habits (via increasing physical activity, maintaining appropriate body weight and healthy nutrition habits, reducing sodium intake, increasing potassium intake from food, no smoking, and no alcohol consumption) are essential in preventing HBP.

## Conclusions

The results of this study showed that both anthropometric parameters – BMI and WC (but especially BMI) – were more strongly related to prehypertension and hypertension, both separately and combined. In addition, they were superior to WHtR in predicting elevated BP among Lithuanian adolescents aged 12–15 years.

## Materials and Methods

### Study population

More detailed information about the study is presented elsewhere^12^.

This cross-sectional study comprised adolescents aged 12 to 15 years who at the time of the examination (from November 2010 to April 2012) attended gymnasiums or secondary schools in Kaunas city and Kaunas district. The schoolchildren (sixth, seventh, eighth, and ninth grades, aged 12–15 years) of the above-mentioned gymnasiums and schools (n = 81) were selected using a two-stage technique. The first stage of sampling involved all gymnasiums and schools of Kaunas city and Kaunas district with adolescents aged 12–15 years. The second stage consisted of the sampling of all grades 6–9 of all the participating schools. Details of the sampling methods have been described previously^71^.

Exclusion criteria included the presence of congenital heart defects, cardiovascular diseases, endocrine diseases, and kidney diseases based on data from medical records. Of 7,638 subjects who participated and were examined in the present study, 152 were excluded due to the above-mentioned diseases. In addition, 29 subjects were excluded from the analysis due to missing anthropometric data. Thus, after the noted exclusion, a total of 7,457 participants were included in the statistical analysis.

The study was approved by Kaunas Regional Ethics Committee for Biomedical Research at the Lithuanian University of Health Sciences (protocol No. BE-2-69). A written informed consent was obtained from each participant’s parent or guardian. All methods were applied in accordance with relevant guidelines and regulations.

### Blood pressure measurements

Details of the measurement methods have been described previously^12^.

Blood pressure was measured in the morning hours by the physician (without wearing a white coat). Before the BP measurement, the subjects were instructed to sit quietly for ten minutes. During the measurement, the participant was in a sitting position with the arm placed and supported at the heart level. BP was measured using an automatic BP monitor (OMRON M6; OMRON HEALTHCARE CO., LTD, Kyoto, Japan) with the appropriate cuff size. BP was measured three times at 5-minute rest intervals between the measurements. The mean of three BP measurements was calculated. Schoolchildren who had an elevated BP (greater than or equal to the 90^th^ percentile) during the first screening underwent a second BP measurement within the period of two–three weeks.

According to BP charts for age, sex, and height, based on the data of “The Fourth Report on the Diagnosis, Evaluation, and Treatment of High Blood Pressure in Children and Adolescents”^69^, NBP was defined as BP < 90^th^ percentile; prehypertension was defined as BP between the ≥90^th^ percentile and the <95^th^ percentile; and hypertension was defined as BP ≥ 95^th^ percentile. The mean arterial pressure (MAP) was calculated using the traditional formula^72^. The pulse pressure (PP) was calculated as SBP minus DBP.

### Anthropometric measurements

The body weight of the subjects (wearing only light indoor clothing and barefooted) was measured to the nearest 0.1 kg with a balance beam scale (SECA). The height of the subjects (without shoes) was measured to the nearest 0.1 cm with a portable stadiometer. BMI was calculated as weight divided by height squared. WC was measured with a flexible measuring tape at a level midway between the lower rib margin and the iliac crest to the nearest 0.5 cm. WHtR was calculated as the WC divided by body height. The Body Roundness index (BRI) was calculated using the following formula^73^:

$$BRI=364.2-365.5\times \sqrt{1-(\frac{{(WC/(2\pi ))}^{2}}{{(0.5\times height)}^{2}})}.$$

### Statistical analysis

We performed statistical analyses using the statistical software package SPSS version 20 for Windows. P < 0.05 was considered statistically significant. Categorical variables were tested by the chi-squared (χ^2^) test, and were expressed as numbers and percentages. The Kolmogorov-Smirnov test was used to test the normality of the distribution of the continuous variables. Non-normally distributed continuous variables were compared using nonparametric tests (the Mann-Whitney U test, and the Kruskal-Wallis test), and were presented as medians and interquartile ranges (25th–75th percentiles). BMI, WC, and WHtR values were converted to age- and sex-specific z-scores. Pearson’s correlation coefficients were calculated between anthropometric indices z-score and SBP, DBP, MAP, and PP. Quartiles of anthropometric indices were calculated according to the study subjects’ age and sex (Table 6). Logistic regression analyses were conducted separately for boys and girls to evaluate the associations between the quartiles of anthropometric parameters (BMI, WC, and WHtR) and HBP. Crude and adjusted odds ratios with 95% confidence intervals (CI) were calculated. The logistic regression models were compared using Akaike’s Information Criterion (AIC). The models with the lowest AIC values were selected as the best. Receiver operating characteristic (ROC) curve analysis was used to evaluate the predictive ability of the anthropometric parameters (BMI z-score, WC z-score, and WHtR z-score) for prehypertension, hypertension and prehypertension/hypertension. The value of the area under the curve (AUC) of the ROC curve was determined as described by Swets^74^ and Greiner *et al*.^75^.

**Table 6:** The age- and sex-specific percentiles of BMI, WC, and WHtR in study participants aged 12–15 years.

Sex	Age (years)	n	BMI			WC			WHtR		
			25^th^	50^th^	75^th^	25^th^	50^th^	75^th^	25^th^	50^th^	75^th^
Boys	12	966	16.67	18.26	20.57	61.00	65.00	71.00	0.39	0.42	0.45
	13	845	17.15	18.66	20.76	63.00	66.00	71.75	0.39	0.41	0.43
	14	891	17.96	19.59	21.39	65.00	69.00	73.00	0.38	0.40	0.43
	15	792	18.71	20.17	21.79	67.00	71.00	75.00	0.38	0.40	0.43
Girls	12	1044	16.23	17.97	20.06	59.00	62.00	67.00	0.38	0.40	0.42
	13	1027	17.16	18.80	20.95	60.00	64.00	68.00	0.37	0.39	0.42
	14	1042	17.64	19.29	21.11	61.0	64.00	69.00	0.37	0.39	0.42
	15	850	18.35	19.81	21.79	62.00	65.00	69.00	0.37	0.39	0.42

BMI – body mass index, WC – waist circumference, WHtR – waist-to-height ratio.

## Data Availability

According to the Statute of the Lithuanian University of Health Sciences, the authors cannot share the data underlying this study. For inquires on the data, researchers should first contact the owner of the database, the Lithuanian University of Health Sciences.
